# Comparison of clinical features and prognostic factors in HIV-negative adults with cryptococcal meningitis and tuberculous meningitis: a retrospective study

**DOI:** 10.1186/s12879-016-2126-6

**Published:** 2017-01-10

**Authors:** Junyan Qu, Taoyou Zhou, Cejun Zhong, Rong Deng, Xiaoju Lü

**Affiliations:** Center of Infectious Disease, West China Hospital, Sichuan University, 37 Guoxue Lane, Chengdu, 610041 China

**Keywords:** Cryptococcal meningitis, Tuberculous meningitis, Clinical features, Prognostic factors, HIV negative

## Abstract

**Background:**

The incidence of cryptococcal meningitis (CM) and tuberculous meningitis (TBM) have gradually increased in recent years. These two types of meningitis are easily misdiagnosed which leads to a poor prognosis. In this study we compared differences of clinical features and prognostic factors in non-HIV adults with CM and TBM.

**Methods:**

We retrospectively reviewed the medical records of CM and TBM patients from January 2008 to December 2015 in our university hospital in China. The data included demographic characteristics, laboratory results, imaging findings, clinical outcomes.

**Results:**

A total of 126 CM and 105 TBM patients were included. CM patients were more likely to present with headache, abnormal vision and hearing, and they might be less prone to fever and cough than TBM patients (*P* < 0.05). Higher percentage of CM patients presented with cerebral ischemia/infarction and demyelination in brain MRI than TBM patients (*P* < 0.05). CM patients had lower counts of WBC in CSF, lower total protein in CSF and serum CD4/CD8 ratio than TBM patients (*P* < 0.05). After three months of treatment, CM group have worse outcome than TBM group (*P* < 0.05). Multivariate analysis showed that age more than 60y (OR = 4.981, 95% CI: 1.955–12.692, *P* = 0.001), altered mentation (OR = 5.054, 95% CI: 1.592–16.046, *P* = 0.006), CD4/CD8 ratios < 1 (OR = 8.782, 95% CI: 2.436–31.661, *P* = 0.001) and CSF CrAg ≥ 1:1024 (OR = 4.853, 95% CI: 1.377–17.098, *P* = 0.014) were independent risk factors for poor prognosis for CM patients. For TBM patients, hydrocephalus (OR = 7.290, 95% CI: 1.630–32.606, *P* = 0.009) and no less than three underlying diseases (OR = 6.899, 95% CI: 1.766–26.949, *P* = 0.005) were independent risk factors, headache was a protective factor of prognosis.

**Conclusions:**

Our study provided some helpful clues in the differential diagnosis of non-HIV patients with CM or TBM and identified some risk factors for the poor prognosis of these two meningitis which could help to improve the treatment outcome. Further studies are worth to be done.

**Electronic supplementary material:**

The online version of this article (doi:10.1186/s12879-016-2126-6) contains supplementary material, which is available to authorized users.

## Background

Many organisms such as *meningococcus*, *Mycobacterium tuberculosis* and *Cryptococcus neoformans* can result in meningitis. With increasing use of immune suppressive medication use in rheumatologic conditions, oncology, and organ transplantation, the incidence of Cryptococcal meningitis (CM) and tuberculous meningitis (TBM) are rising [1, 2].

CM and TBM are the two most common types of chronic infectious meningitis, especially in developing countries, and may have similar clinical manifestations and cerebrospinal fluid (CSF) findings [3, 4]. Although mortality has decreased significantly in recent years, with the advent of new antimicrobial drugs, diagnostic techniques and treatment strategies, it remains high [3, 5–7]. CM and TBM are easily misdiagnosed due to vague clinical syndromes associated with these conditions [3, 6]. Delay of diagnosis and treatment are directly related to a poor prognosis [3, 8]. Early diagnosis CM and TBM, especially TBM, remain challenges for clinicians.

CM and TBM are common in human immunodeficiency virus (HIV) infected patients, but they are also seen in patients with other forms of immunosuppression and apparently immunocompetent individuals [9]. Some studies have sought to identify clinical features in order to distinguish TBM from CM in human HIV infected patients [10, 11]. Data on how to identify CM and TBM according to clinical profiles in HIV negative patients are scarce. In this retrospective study, we aimed to compare the demographic features, clinical presentations, laboratory data, radiographic findings, therapeutic outcomes and prognostic factors of HIV negative TBM and CM patients who were admitted to a university hospital from 2008 to 2015 in China.

## Methods

### Study population

The retrospective study was conducted between January 2008 and December 2015, in West China Hospital, Sichuan University, China (a 4,300-bed academic tertiary care hospital). Patients aged 14 years or older with confirmed diagnosis of CM or clinical or confirmed diagnosis of TBM were included. Exclusion criteria were age under 14 years, HIV positive with infected, pregnant mothers, CM combined with TBM, and cases lacking follow-up for anti-tuberculosis or anti-fungal treatment.

The diagnosis of CM and TBM were made by clinical and laboratory findings. Symptoms and signs of meningitis including one or more of the following: headache, vomiting, fever, neck stiffness, convulsions, focal neurological deficits, altered mentation. Laboratory assessments for CM included CSF culture, CSF India ink testing, and CSF cryptococcal antigen testing (CrAg). Patients with TBM included definite TBM and clinical diagnosis of TBM. The diagnostic criteria of definite TBM was clinical findings plus one or more of the following: acid-fast bacilli seen in the CSF, *M. tuberculosis* cultured from the CSF, or a CSF positive commercial nucleic acid amplification test. The clinical diagnosis of TBM based on the clinical algorithm [12], which modified according to Marais’ definitions [13]. All enrolled patients were divided into CM and TBM groups.

Altered mentation in CM and TBM refers to general changes in brain function, such as confusion, amnesia, loss of alertness, loss of orientation, defects in judgment and unusual behavior. Which were confirmed by psychiatrists.

### Laboratory studies

Laboratory tests such as complete blood counts, blood biochemistry, interferon gamma release assays (IGRAs), peripheral blood T lymphocyte subset measurements and HIV test were done at the department of laboratory medicine of our hospital. Lumbar puncture (LP) and tuberculin skin test (TST) of suspected CM or TBM cases were performed before initial treatment. CSF was sent for WBC, glucose, protein, chloride, India ink stain, CSF cryptococcal antigen titers, TB smear, TB PCR, fungal and *M. tuberculosis* cultures. These tests were done at the discretion of the treating physicians. Fluorescence quantitative PCR was used to detect TB DNA in CSF. The rapid liquid culture mediums were used to detect *M. Tuberculosis*. Fungal culture of CSF was performed on Sabouraud dextrose agar (SDA) and incubated at 30°C to obtain cryptococcus isolates. The CSF cryptococcal antigen titers were determined by Latex Cryptococcal Antigen Detection System (Immuno-Mycologics, Inc., Norman, USA). The test utilizes latex particles coated with anti-cryptococcal antibody, which will reacts with the cryptococcal capsular polysaccharide antigen causing a visible agglutination. Brain magnetic resonance imaging (MRI) or computed tomography (CT) examinations and chest CT were performed at the discretion of the physicians.

### Treatment strategies

All the patients received treatments after they got the diagnoses. Time of diagnosis was the time from onset of symptoms to treatment. HIV negative CM patients were treated with amphotericin B (0.7–1 mg/kg/d), 5-flucytosine (100 mg/kg/d) with or without fluconazole (800 mg/day)/voriconazole (loading dose, 6mg/kg/12h, maintenance dose, 4mg/kg/12h) for 6–12 weeks of induction therapy, and then fluconazole (400 mg/day) for 8 weeks of consolidation therapy, fluconazole (200 mg/day) for more than 6 months of maintenance therapy according to the guidelines for the management of cryptococcal disease and our experiences [5, 14]. Only three patients with CM received voriconazole. Because amphotericin B was contraindicated in some patients with kidney disease or renal inadequacy emerged after use of amphotericin B in another patient. Continuous lumbar drainage subarachnoid were applied to the CM patients with persistent high intracranial pressure. The treatment regimen for TBM patients who had not been treated with chemotherapy consisted of three months of isoniazide (INH), rifampin (RIF), pyrazinamide (PZA), and streptomycin (SM)/ethambutol (EMB), followed by INH and RIF [15, 16]. The total treatment course was at least 12 month. Second-line treatment regimens were given after failure to first-line TB chemotherapy according to the guideline and patients’ situation [15, 16].

### Data collection

We retrospectively reviewed the medical records of patients involved in this study. We obtained demographic characteristics, underlying diseases, use of steroids, symptoms and signs on admission, duration of initial symptoms to diagnosis or treatment, laboratory data, brain MRI /CT findings and clinical outcomes. The clinical outcomes were evaluated after three-month of treatment. Risk factors for the prediction of poor of HIV negative CM and TBM patients were also evaluated. All data were verified by two authors independently.

### Ethical considerations

The study was approved by the Ethics Committee of West China Hospital, Sichuan University, which waived the need for informed consent because all the data used in this study were routinely obtained and no additional procedures were carried out.

### Statistical analysis

Statistical analyses were performed using SPSS version 17.0 software package (SPSS Inc., Chicago, USA). The Shapiro-Wilk normality test was used for testing normality for all quantitative variables. Continuous variables were presented as mean ± standard deviation or median with interquartile ranges (IQR). Signifcance testing was carried out using Student’s *t* test for normally distributed data and Mann-Whitney *U* test for non-normal data. Chi-square test or Fisher exact test was used for categorical variables. Risk factors for three-month treatment outcomes of CM or TBM were identified using a logistic regression model. All variables were initially tested by univariate analysis and those with a *P* < 0.05 were entered stepwise into multivariate analysis by using the forward conditional method. Then the regression equations for predicting the probability of poor prognosis of CM or TBM were established. Receiver operating characteristic (ROC) curves were plotted for these two predicted models with the Medcalc software version 11.5.1.0 (Medcalc software bvba, Belgium). Predictive accuracy was assessed by calculating the areas under the ROC curves (AUC). An AUC of > 0.8 was considered very good. A two-tailed *P* < 0.05 was considered statistically significant.

## Results

### Patient characteristics

From January 2008 to December 2015, a total of 126 CM patients and 105 TBM patients without HIV infection were included in this retrospective study. The clinical characteristics of these patients were shown in Table 1. There was no significant difference between CM and TBM patients in gender.

**Table 1:** Demographic and clinical features in HIV negative patients with cryptococcal meningitis (CM) and tuberculous meningitis (TBM)

Variables	CM (*N*=126)	TBM (*N*=105)	*P*-value
Sex			
Male	79(62.7%)	57(54.3%)	0.227
Female	47(37.3%)	48(45.7%)	
Age (years)			
≤30	29(23.0%)	54(51.4%)	**<0.001**
31–60	65(51.6%)	35(33.3%)	
>60	32(25.4%)	16(15.2%)	
Presenting symptoms and signs			
Headache	118(96.7%)	87(82.9%)	**0.012**
Fever	71(56.3%)	72(68.6%)	**0.038**
Vomiting	71(56.3%)	47(44.8%)	0.263
Altered mentation	51(40.5%)	43(41.0%)	0.524
Abnormal vision	35(27.8%)	12(11.4%)	**0.002**
Cough	7(5.6%)	24(22.9%)	**<0.001**
Seizure	14(11.1%)	6(5.7%)	0.111
Abnormal hearing	14(11.1%)	3(2.9%)	**0.014**
Apopsychia	6(4.8%)	2(1.9%)	0.208
Dyspnea	5(4.0%)	1(1.0%)	0.155
Meningeal signs	89(70.6%)	68(64.8%)	0.209
Pathological signs	22(17.5%)	23(21.9%)	0.247
Underlying diseases, factors or complications			
Corticosteroid medication	22(17.5%)	6(5.7%)	**0.005**
Pnumonary infection	22(17.5%)	13(12.4%)	0.188
Hepatobiliary diseases	15(11.9%)	12(11.4%)	0.539
Tuberculosis outside CNS	12(9.5%)	62(59.0%)	**<0.001**
Immune system disease	11(8.7%)	6(5.7%)	0.269
Hypertension	11(8.7%)	3(2.9%)	0.054
Diabetes mellitus	13(10.3%)	10(9.5%)	0.510
Chronic lung disease	6(4.8%)	3(2.9%)	0.348
Multiple organ dysfunction syndrome	6(4.8%)	1(1.0%)	0.095
Renal transplantation	4(3.2%)	2(1.9%)	0.431
Hematology disease	3(2.4%)	1(1.0%)	0.382
Others	13(10.2%)	12(11.4%)	0.571
None	29(23.0%)	20(19.0%)	0.284
Time from onset of symptoms to treatment (d)			
0–30	48(38.1%)	56(53.3%)	**<0.001**
31–60	47(37.3%)	33(31.4%)	
61–90	19(15.1%)	13(12.4%)	
>90	12(9.5%)	3(2.9%)	
Outcome Improved	80(63.5%)	81(77.1%)	0.031
Worse/Died	46(36.5%)	24(22.9%)	

Most patients with CM were from 30 to 60 years (65/126, 51.6%), but most of TBM patients were aged under 30 years (54/105, 51.4%). Among CM patients, the most common initial symptoms were headache (118/126, 96.7%), followed by fever (71/126, 56.3%), vomiting (71/126, 56.3%) and altered mentation (51/126, 40.5%). The most common presenting symptoms of TBM patients were also headache (87/105, 82.9%), fever (72/105, 68.6%) and vomiting (47/105, 44.8%). CM patients were more likely to present with headache, abnormal vision and hearing, and less likely to present with fever and cough than TBM patients (*P* < 0.05).

The most frequently underlying diseases, factors or complications of CM patients were corticosteroid use (22/126, 17.5%), pulmonary infection (22/126, 17.5%), hepatobiliary diseases (15/126, 11.9%) and diabetes mellitus (13/126, 10.3%). However, tuberculosis outside CNS was the most common in TBM patients, especially disseminated tuberculosis (27/62, 43.5%, data not shown). Twenty percent of patients had no underlying condition.

### Laboratory data and radiological findings

Laboratory data and radiographic findings are prior to shown in Table 2. The CSF WBC and total protein in CM patients were lower than those of TBM group (*P* < 0.05). There was no statistical difference in CSF glucose, chloride level and opening pressure in LP between the two groups. In HIV negative CM group, CD4/CD8 ratio in serum was significantly decreased compared to the TBM group.

**Table 2:** Laboratory data and radiographic findings in HIV negative patients with cryptococcal meningitis (CM) and tuberculous meningitis (TBM)

Variables	CM (*N*=126)	TBM (*N*=105)	*P*-value
CSF			
WBC (cells/mm^3^), Median (IQR)	80.00 (20.00–200.00)	120.00 (59.00–295.00)	**0.005**
Total protein (mg/dL), Median (IQR)	0.83 (0.58–1.32)	1.53(0.99–2.66)	**<0.001**
Glucose (mmol/L), Median (IQR)	1.57 (0.80–2.56)	1.78(1.10–2.35)	0.694
Chloride (mmol/L), (mean±SD)	118.11±6.67	111.40±7.59	0.169
Open pressure > 220mm H_2_O	98 (77.78%)	75 (71.4%)	0.289
Serum			
Albumin(g/L), (mean±SD)	37.77±5.29	36.61±5.20	0.688
WBC (×10^9^/L), (mean±SD)	8.89±3.51	7.46±3.45	0.701
Neutrophile (%)	78.44±10.85	75.92±11.33	0.690
Hemoglobin (g/L), (mean±SD)	122.83±23.05	118.50±22.71	0.548
CD3^+^ T lymphocyte (%)	65.11±14.25	70.96±13.20	0.533
CD4^+^ T lymphocyte (%)	28.71±10.46	34.77±11.27	0.542
CD8^+^ T lymphocyte (%)	25.40±8.57	25.19±10.26	0.139
CD4/CD8 ratio	1.22±0.59	1.71±1.12	**0.003**
Brain images (CT/MRI)			
Cerebral ischemia/infarction	55 (43.7%)	30 (28.6%)	**0.028**
Demyelination	30 (23.8%)	14 (9.5%)	**0.046**
Meningeal enhancement	23 (18.3%)	20 (16.0%)	0.877
Hydrocephalus	16 (12.7%)	17(16.2%)	0.709
Encephalitis	14 (11.1%)	40 (38.1%)	**<0.001**
Cerebral edema	4 (3.17%)	13 (12.4%)	**0.010**
Others	4 (3.17%)	8 (7.6%)	0.147
Normal	20 (15.9%)	12 (11.4%)	0.347

*CSF* cerebrospinal fluid, *WBC* white blood count, *MRI* magnetic resonance imaging

Brain MRI was performed in 84.9% (107/126) patients with CM and 76.2% (80/105) patients with TBM. Other patients were examined by head CT. The most common manifestations were cerebral ischemia, demyelination, meningeal enhancement and encephalitis in these two groups. A total of 15.9% (20/126) patients in CM group and 11.4% (12/105) in TBM group had normal image findings. A higher percentage of CM patients presented with cerebral ischemia/infarction and demyelination than TBM patients (*P* < 0.05), but encephalitis and cerebral edema were more common in TBM patients (*P* < 0.05).

CM and TBM specific test results are shown in Table 3. For CM patients, latex agglutination CrAg titer were positive in 99.2% (125/126) of them. The positive rates of CSF India ink stain and CSF culture for *C. neoformans* in CM patients were 77.8% (98/126) and 79.3% (100/126), respectively. IGRA had higher positive rate than other detection methods in TBM patients (*P* < 0.05).

**Table 3:** The positive rate of pathogens and other serological tests for diagnosis of cryptococcal meningitis (CM) and tuberculous meningitis (TBM) in HIV negative patients

Variables	CM (*N*=126)	Variables	TBM (*N*=105)
India ink stain	98 (77.8%)	TB smearing	5 (4.8%)
CSF fungal culture	100 (79.3%)	TB DNA	59 (56.2%)
India ink stain & fungal culture	72 (57.1%)	Mycobacterium tuberculosis culture	9 (8.6%)
CrAg	125 (99.2%)	IGRA	94 (89.5%)
CrAg titer ≥1:1024	73 (57.9%)	TST	62 (60.0%)

*CSF* cerebrospinal fluid, *CrAg* cryptococcal antigen, *TST* tuberculin skin test, *IGRA* interferon gamma release assay

### Treatment outcome

A total of 38.1 and 53.3% of patients with CM and TBM received treatment within 30 days after the onset of clinical symptoms, respectively. And the proportion of patients with a duration of more than 2 month was higher in the CM group than TBM group (26.6% vs. 15.2%, *P* = 0.100), but there was no significant difference. After three months of treatment, 63.5% (80/126) of CM patients and 77.1% (81/105) of TBM patients had improved (*P* = 0.031).

### Risk factors for poor treatment outcome of HIV negative CM and TBM patients

Univariate analysis revealed that the factors impacting the prognosis of CM patients were age, altered mentation, demyelination, CD4/CD8 ratios < 1 and CSF CrAg ≥ 1:1024. Multivariate analysis showed that age, altered mentation, CD4/CD8 ratios < 1 and CSF CrAg ≥ 1:1024 were independent risk factors for poor prognosis. For TBM patiens, multiple regression analysis showed that hydrocephalus (OR = 7.290, 95% CI: 1.630–32.606, *P* =0.009) and no less than three underlying diseases (OR = 6.899, 95% CI: 1.766–26.949, *P* = 0.005) were the risk factors, and headache was a protective factor of prognosis, as shown in Table 4.

**Table 4:** Risk factors associated with poor prognosis of HIV negative cryptococcal meningitis (CM) and tuberculous meningitis (TBM) patients

Cryptococcal meningitis (CM)						
Variables	Univariate analysis			Multivariate analysis		
	Odds ratio	95% CI	*P*-value	Odds ratio	95% CI	*P*-value
Ages>60y	5.946	2.493–14.183	<0.001	4.981	1.955–12.692	0.001
Altered mentation	4.533	1.763–11.656	0.002	5.054	1.592–16.046	0.006
Demyelination	7.270	2.560–20.646	<0.001			
CD4/CD8 ratio<1	8.475	2.831–25.378	<0.001	8.782	2.436–31.661	0.001
CrAg titer ≥1:1024	5.338	1.769–16.103	0.003	4.853	1.377–17.098	0.014
Tuberculous meningitis (TBM)						
Fever	9.659	1.208–77.252	0.033			
Headache	0.139	0.042–0. 459	0.001	0.125	0.032–0.497	0.003
Hydrocephalus	3.692	1.062–12.839	0.040	7.290	1.630–32.606	0.009
Underlying diseases or factors≥3	6.817	1.927–19.869	0.002	6.899	1.766–26.949	0.005

*HIV* human immunodeficiency virus, *CrAg* cryptococcal antigen, *CI* confidential intervals

### ROC curve for the prognostic index derived from the logistic regression models

Prediction models for the probability of poor prognosis in patients with CM and TBM are shown in Appendix 1. The ROC curves of the models of CM and TBM are shown in Additional file 1: Figure S1.

## Discussion

Many patients with CM and TBM were misdiagnosed or had a delay in diagnosis. Fever, headache, vomiting, altered mentation and meningeal irritation were the most common initial symptoms of both CM and TBM patients. However, CM patients were more likely to suffer headache, vision and hearing damage, TBM patients were more prone to have fever and cough. These results were fairly consistent with previous reports [9, 17, 18]. One review showed headache (87%), fever (74%), meningeal irritation (67%), vomiting (61%) and altered mentation (26%) as the most common clinical manifestations of CM [9]. One study showed that among patients without HIV, those with CM were more likely to present headache, altered mentation, vision and hearing damage as compared to those with TBM [18].

Although CM and TBM are often considered as an opportunistic infection in patients with HIV, they are also seen in individuals with apparently normal immune function. In this study, corticosteroid medication, hepatobiliary diseases (abnormal liver function for various reasons, such as chronic hepatitis, cirrhosis) and diabetes mellitus occupied the top three underlying diseases and factors for CM patients. A study reviewed 3698 cryptococcosis cases that found the most common underlying diseases were HIV infection, tuberculosis, liver disease, systemic lupus erythematosus and diabetes mellitus, while 17% had no underlying diseases [9]. Another study showed the commom underlying disease of HIV negative CM patients were hepatobiliary diseases, hypertension, cancer and diabetes mellitus [7]. Sixty-two TBM patients, 27 of them were disseminated tuberculosis, in this study were known to have pulmonary tuberculosis. Gu et al. also found 30.1% of TBM patients derived their TBM from disseminated tuberculosis [17]. TBM is the most severe form of extrapulmonary tuberculosis, it can occur in isolation or along with a pulmonary focus. TBM and disseminated tuberculosis are usually due to hematogenous dissemination of the tubercle bacillus [19].

In CM and TBM patients, the most common manifestations on brain imagines were cerebral ischemia/infarction, meningeal enhancement, hydrocephalus, which consistent with previous reports [3, 5, 20]. Demyelination were seen on some patients’ head MRI or CT. Of course, MR imaging is more sensitive in depicting demyelination than head CT. In this study, demyelination was more commom for CM patients than in prior reports [5, 20]. Another study showed that gadolinium-enhancing white matter lesions found on brain MRI of CM patients. Biopsy revealed the lesions of resembled demyelinating were cryptococci, non-specific inflammatory changes or small perivascular lymphocyte collections. Which possibly be leukoencephalopathy or immune response to the pathogen [21]. In addition, our patients showed evidence of cerebral ischemia or encephalitis more often than previous studies [9, 18], this was likely related to the increased sensitivity of MRI with diffusion-weighted imaging.

Patients with TBM presented with higher CSF WBC and total protein than CM patients, similar to prior studies [18]. A low ratio of CD4/CD8 was also found in the apparently healthy CM patients, it’s possible those patients may have some undetectable underlying disease.

In our study, multivariate analysis showed that age >60 years, altered mentation, CD4/CD8 ratios < 1 and CSF CrAg ≥ 1:1024 were closely correlated with poor outcome of CM patients. Previous studies showed there were many poor prognostic factors for CM, including Cryptococcus growth from sites other than CSF, altered mentation, intracranial pressure (ICP) >400 mmH_2_O, CSF glucose < 40 mg/dl, high CSF CrAg titer, decreased CSF/blood glucose ratio, papilledema, chronic use of corticosteroids or other immunosuppressants, absence of headache and low GCS score [7, 20, 22–24]. Our study showed that CrAg test was highly specific to the diagnosis of cryptococcal meningitis, and it also had prognostic value, similar to previous studies [25]. Though middle-aged people made up a greater percentage of our CM patients, mortality was increased with patients aged greater than than 60 years, possibly related to a decreased immune response to pathogens declines with age [26].

For TBM patients, poor outcome was related to hydrocephalus and more than three underlying diseases in the present study. Hydrocephalus, optochiasmatic arachnoiditis, microbiologically confirmed TBM have been associated with poor outcome of TBM in previous studies [27, 28]. Early diagnosis and treatment improve prognosis. We found no literature showing that more than three underlying diseases was correlated with poor outcome of TBM. It might be because these studies did not include this factor. On admission, there were 17.1% TBM patients had no headache. Headache was a protective factor for TBM in this study. The inflammation that occurs in the subarachnoid space during meningitis can largely be attributed to the response of the immune system to bacteria, which leads to headache [29]. Patients without headache probably means they have weak immune response. So they had poor outcome.

There were some limitations concerning this study. Because our study was a retrospective research, the medical records about epidemiological contact history were incomplete, so we did not analyse relevant contents. In addition, there were three patients with CM using voriconazole because they could not tolerate the adverse effect of amphotericin B or they complicated renal disease. But just three patients used non standard treatments, which might not affect the statistic results.

## Conclusions

We found that compared to patients with TBM, CM patients were more likely to present with headache, vision and hearing damage, cerebral ischemia and demyelination. TBM patients were more likely to have fever, cough, encephalitis and cerebral edema. CM patients had lower CSF WBC, total protein, and CD4/CD8 ratio than those of TBM patients. Prognosis was poorer for CM patients than TBM patients. Age, altered mentation, CD4/CD8 ratios < 1 and CSF CrAg ≥ 1:1024 were independent risk factors for poor prognosis of HIV negative CM patients. Hydrocephalus and no less than three underlying diseases were the risk factors, and headache was a protective factor for HIV negative patients with TBM. Our prediction models may be helpful for making treatment plan and prognosis assessment. Of course, specific identification of the offending microorganism is considered to be the gold standard in diagnosing infectious diseases. More work needs to be done to develop more rapid, sensitive and specific diagnosis methods and more effective treatments for patients CM and TBM, and the prognosis can be improved accordingly.

## Appendix 1

### Prediction models for the probability of poor prognosis in patients with cryptococcal meningitis (CM) and tuberculous meningitis (TBM)

Prediction model for the probability of poor prognosis in CM: *P*_*CM*_=e^x^/(1 + e^x^), x = −6.360 + (1.606 × age) + (1.620 × altered mentation) + (2.173 × CD4/CD8 ratios) + (1.579 × CSF CrAg titer). Prediction model for the probability of poor prognosis in TBM: *P*_*TBM*_=e^x^/(1 + e^x^), x = −0.658 + (1.931 × underlying diseases) + (1.987 × hydrocephalus) + (−2.079 × headache). Where the following are used: *e* is the natural logarithm; age>60y, altered mentation, CD4/CD8 ratios<1, CSF CrAg titer ≥1:1024, underlying diseases≥3 and headache are scored as 1 or otherwise as 0; hydrocephalus was derived from the imaging report (1: yes, 0: no). The ROC curves of the risk index models for predicting poor prognosis of CM or TBM are shown in Additional file 1: Figure S1. The AUC of CM was 0.872 (95% CI: 0.786–0.932). The appropriate cut off point was selected at P_CM_=0.5127, and the model achieved a sensitivity of 69.44%, a specificity of 91.23%. The AUC of TBM was 0.802 (95% CI: 0.700–0.882), the appropriate cut off point was 0.3209 for P_TBM_ with a sensitivity of 57.89% and a specificity of 88.89%.

## Additional file

Additional file 1: Figure S1. Prediction models for the probability of poor prognosis in patients with cryptococcal meningitis and tuberculous meningitis. Receiver operator characteristic (ROC) curve for the prognostic index derived from the logistic regression models of cryptococcal meningitis and tuberculous meningitis. For cryptococcal meningitis patients, area under the ROC curve = 0.872 (95% CI: 0.786–0.932), for tubercular meningitis patients, area under the ROC curve = 0.802 (95% CI: 0.700–0.882). (TIF 3370 kb)

## Abbreviations

5-FC: 5-flucytosine
AIDS: Acquired immune deficiency syndrome
AMB: Amphotericin B
CM: Cryptococcal meningitis
CNS: Central nervous system
CrAg: Cryptococcal antigen
CSF: Cerebrospinal fluid
CT: Computed tomography
EMB: Ethambutol
HIV: Human immunodeficiency virus
IGRA: Interferon gamma release assay
INH: Isoniazide
LP: Lumbar puncture
MRI: Magnetic resonance imaging
PCR: Polymerase chain reaction
PZA: Pyrazinamide
RIF: Rifampin
TBM: Tuberculous meningitis
TST: Tuberculin skin test
WBC: White blood cell count
