# Supplementary figures and images for: Comparison of clinical features and prognostic factors in HIV-negative adults with cryptococcal meningitis and tuberculous meningitis: a retrospective study
Source: BMC Infect Dis. 2017 Jan 10;17:51. doi: 10.1186/s12879-016-2126-6 (PMC5223460; doi:10.1186/s12879-016-2126-6)

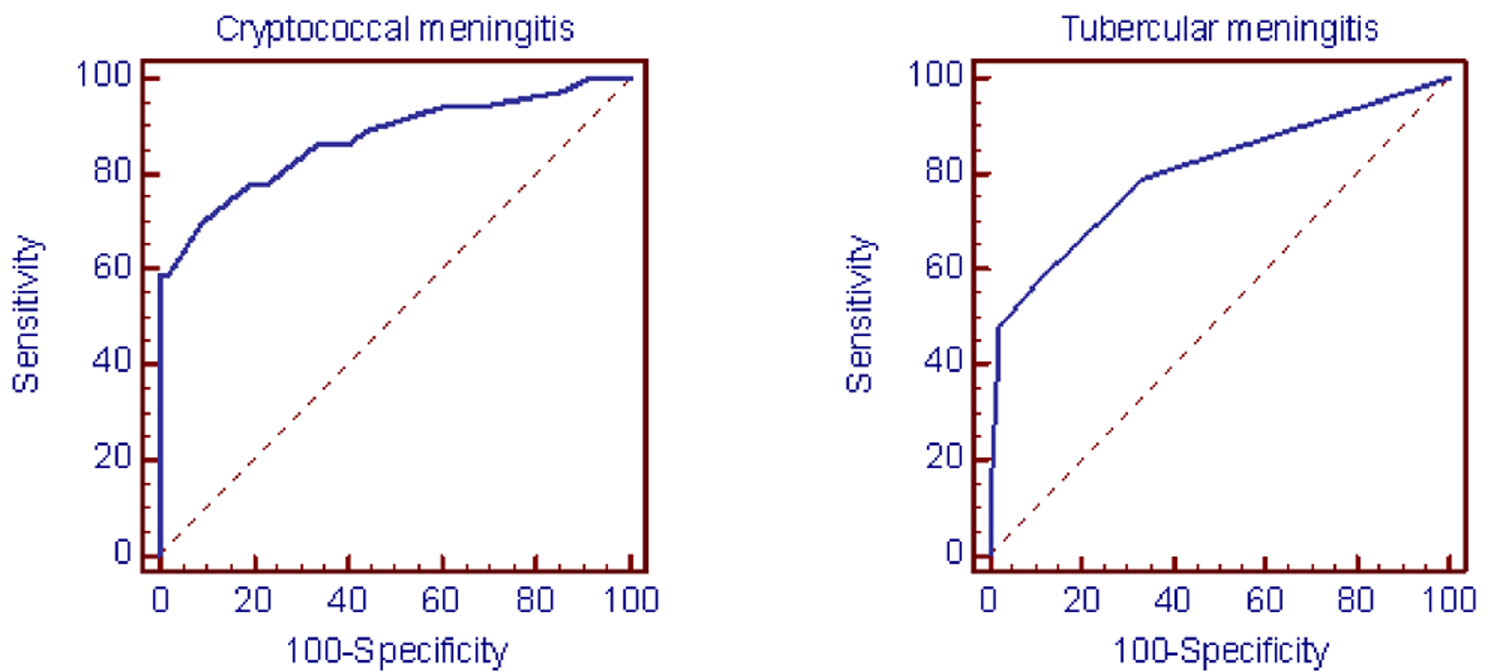

Supplement: Additional file 1: Figure S1. — Prediction models for the probability of poor prognosis in patients with cryptococcal meningitis and tuberculous meningitis. Receiver operator characteristic (ROC) curve for the prognostic index derived from the logistic regression models of cryptococcal meningitis and tuberculous meningitis. For cryptococcal meningitis patients, area under the ROC curve = 0.872 (95% CI: 0.786–0.932), for tubercular meningitis patients, area under the ROC curve = 0.802 (95% CI: 0.700–0.882). (TIF 3370 kb) [file 12879_2016_2126_MOESM1_ESM.tif]
